# A resource from 3D electron microscopy of hippocampal neuropil for user training and tool development

**DOI:** 10.1038/sdata.2015.46

**Published:** 2015-09-01

**Authors:** Kristen M. Harris, Josef Spacek, Maria Elizabeth Bell, Patrick H. Parker, Laurence F. Lindsey, Alexander D. Baden, Joshua T. Vogelstein, Randal Burns

**Affiliations:** 1 Department of Neuroscience, Center for Learning and Memory, Institute for Neuroscience, University of Texas at Austin, 1 University Station C7000, Austin, Texas 78712, USA; 2 Department of Pathology, Charles University at Prague, Faculty of Medicine, 500 35 Hradec Kralove, Czech Republic; 3 Department of Computer Science, Institute for Data Intensive Science and Engineering, Johns Hopkins University, 160 Malone Hall, 3400 N. Charles St., Baltimore, Maryland 21218, USA; 4 Department of Biomedical Engineering, Institute for Computational Medicine, Johns Hopkins University, Clark Hall Room 317C, 3400 N. Charles St., Baltimore, Maryland 21218, USA

**Keywords:** Neuroinformatics, Neuroscience

## Abstract

Resurgent interest in synaptic circuitry and plasticity has emphasized the importance of 3D reconstruction from serial section electron microscopy (3DEM). Three volumes of hippocampal CA1 neuropil from adult rat were imaged at X-Y resolution of ~2 nm on serial sections of ~50–60 nm thickness. These are the first densely reconstructed hippocampal volumes. All axons, dendrites, glia, and synapses were reconstructed in a cube (~10 μm^3^) surrounding a large dendritic spine, a cylinder (~43 μm^3^) surrounding an oblique dendritic segment (3.4 μm long), and a parallelepiped (~178 μm^3^) surrounding an apical dendritic segment (4.9 μm long). The data provide standards for identifying ultrastructural objects in 3DEM, realistic reconstructions for modeling biophysical properties of synaptic transmission, and a test bed for enhancing reconstruction tools. Representative synapses are quantified from varying section planes, and microtubules, polyribosomes, smooth endoplasmic reticulum, and endosomes are identified and reconstructed in a subset of dendrites. The original images, traces, and Reconstruct software and files are freely available and visualized at the Open Connectome Project (Data Citation 1).

## Background & Summary

Serial section electron microscopy has long been used to visualize and reconstruct cellular and subcellular components of biological systems^1–10^. Recently, 3DEM has become recognized as an important tool to map and understand synaptic circuitry in the brain^11–15^. Obtaining detailed representations of the structural organization of dendrites, axons, glia, and their subcellular components is fundamental to understanding cellular mechanisms of behavior, learning, and memory^16–20^. Results from 3DEM are beginning to provide crucial insights into the anatomical substrates of information processing and behavioral output in normal human brains and in pathological conditions such as Alzheimer’s disease^21–28^.

Past technologies limited the exploration of EM series to the investigators who collected them and their immediate colleagues. Modern computing and imaging tools now allow the open sharing of this complex data^13,29–34^. Here, we describe three densely reconstructed volumes from adult hippocampal CA1 neuropil that were prepared with the widely used Reconstruct software^35,36^.

Previous findings from these and other hippocampal series illustrate the necessity of 3DEM. Thus, it is important to share the original images and traces to train new users and ultimately to enhance computer algorithms that facilitate automated approaches on these complicated images. From the axonal perspective, we have shown that ~20% of the axons reaching a CA1 dendrite form a synapse with it. Furthermore, the gross axonal or dendritic geometry alone is insufficient to predict synapse locations; hence, they must be identified through 3DEM^11^. Each presynaptic bouton can have zero, one, or multiple postsynaptic partners^37,38^. The number of presynaptic vesicles in an axonal bouton is highly correlated with the associated postsynaptic area, both of which vary more than tenfold in parallel with dendritic spine head dimensions^37,39–42^. Axon-coupled spines from the same dendrite have nearly identical head volumes, whereas all other configurations of axon-spine coupling show no correlation in spine head volume^38^. These findings suggest a highly precise coordination between pre- and postsynaptic structure and function.

Endocytic and exocytic organelles occur in about 20% of CA1 dendritic spines, yet they rarely occur together or with smooth endoplasmic reticulum (SER) in the same spine^43–45^. SER in dendritic shafts forms simple tubules along aspiny segments or complex branched and swollen regions at the base of large dendritic spines, where cargo is slowed and readily off-loaded to support synapses locally^43,46^. The largest spines are more likely to contain multiple organelles and polyribosomes, suggesting that they could respond rapidly to local changes in synaptic efficacy^18,47^. Some synapses have an edge region called the nascent zone that is adjacent to the active zone. This region is likely to be silent because it lacks presynaptic vesicles in close enough proximity to activate putative postsynaptic receptors in the nascent zone^48–51^. Like the neighboring active zone, the nascent zone is structurally dynamic during different stages of synaptic plasticity^51^. The extracellular space is organized into tunnels and sheets that allow for differential diffusion of small and large molecules amongst neuronal and glial processes^52^. Together these features might also account for the preferential distribution of perisynaptic astroglial processes to larger CA1 synapses^53,54^. None of these findings are revealed without 3DEM.

There remain many questions to investigate even in these densely reconstructed volumes, such as: What is the local circuitry of inhibitory axons? How often do mitochondria branch or split? Do vesicles or glycogen granules aggregate in astroglial processes near synapses? What is the natural distribution of autophagosomes? The image volumes go well beyond the reconstructed regions and hence contain portions of these objects that are ideal for instruction as they can be traced back to identified objects.

**Figure 1:**
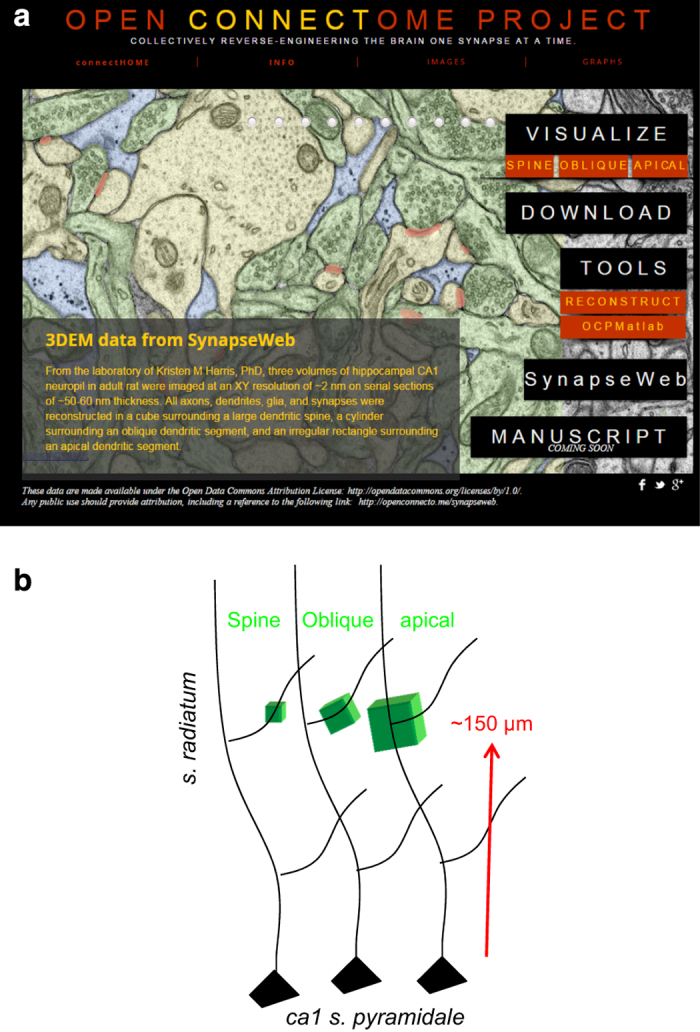
Sharing three volumes of hippocampal neuropil. (**a**) Navigating within the Open Connectome Project (OCP, Data Citation 1). From the Project home page in OCP, the user can click on one of the three volumes under Visualize for viewing in CATMAID. Download provides access to all forms of the data, and a page with detailed descriptions of the underlying data. The Tools tab provides several environments in which the data and images can be investigated after download. The SynapseWeb tab is a direct link to that website with additional tutorials and an online ultrastructural atlas. (**b**) Locations and relative sizes of the three image volumes. Subregions of volumes 1–3 were fully reconstructed in 3DEM as illustrated in subsequent figures and images are available for download from OCP.

Guidelines for object identification and preparing dense reconstructions are presented here to instruct new users of 3DEM in the organization and composition of complex neuropil. The images and ground truth reconstructions can also be used to improve automated segmentation algorithms that should facilitate future investigations in 3DEM^33,55,56^. All three annotated series and associated Reconstruct files are publicly available for viewing and download at the Open Connectome Project (Fig. 1a, Data Citation 1).

## Methods

### Tissue source and processing

All procedures followed NIH guidelines for the humane care and use of laboratory animals. A male rat of the Long-Evans strain weighing 310 grams (postnatal day 77) was perfused through the heart under deep pentobarbital anesthesia with 2% paraformaldehyde, 2.5% glutaraldehyde, and 2 mM CaCl_2_ in 0.1 M cacodylate buffer at pH 7.35, 37 °C, and 4 psi, as previously described^37^. The brain was left undisturbed in the cranium for 1 h, and then the hippocampus was removed. Immediately after dissection, the hippocampus was chopped to 400 μm thickness. The sections were washed with agitation in buffer and then soaked for 1 h in 1% OsO_4_ with 1.5% potassium ferrocyanide followed by 1 h in 1% OsO_4_. Then they were rinsed in buffer, soaked in 30% and 50% ethanol for 10 min each, immersed for 1 h in 1% uranyl acetate in 70% ethanol at room temperature, dehydrated through ethanols and propylene oxide and embedded in Epon and cured for 48 h at 60 °C. Series were cut according to our published protocols^57^. Briefly, a diamond trimming tool (Electron Microscopy Sciences, Fort Washington, PA) was used to prepare small trapezoidal areas ~200 μm wide by 30–50 μm high. Serial thin sections were cut at a setting of ~50 nm on a Reichert Ultracut E ultramicrotome. Serial sections were collected on Formvar or pioloform coated slot grids (Synaptek) and counterstained with saturated ethanolic uranyl acetate followed by Reynolds’ lead citrate, each for 5 min. Individual grids were placed in grid cassettes and stored in numbered gelatin capsules. The custom-made cassettes (previously used for imaging in Harris *et al.* ^57^) were mounted in the JEOL rotating stage to obtain uniform orientation of the sections on adjacent grids. The series were photographed on a JEOL 1200EX or 1230 electron microscope (JEOL, Peabody, MA). Initial magnifications were 5000x–6000x. For future work, we recommend the scanning electron microscope operating in the transmission mode (tSEM), as delineated in our recent publication^58^. Image quality is similar when pixel size of 2 nm or less is maintained. (This resolution is needed to visualize microtubules for quantification, as an independent measure of dendrite caliber needed in our experiments.) Furthermore, much larger image fields can be collected with 2 nm resolution (or less) semi-automatically on the new tSEM system, at great savings both in time and cost to the user^58^.

### Electron microscopy

Three volumes were photographed from the middle of s. radiatum, each ~150–200 μm from the hippocampal CA1 pyramidal cell soma layer (Fig. 1b, Table 1 (available online only)). Throughout this report, each volume is referred to by its central object, i.e. spine, oblique dendrite, or apical dendrite. The 60 images for the spine volume were obtained from digital scans of original EM negatives from series 24 in our prior publication (k24, Table 1 (available online only))^37^. Additional serial sections were cut from a different block from the same animal and two image fields were photographed from these sections (R34CA1-B_S12 refers to Rat 34 CA1, block B, series 12, Table 1(available online only)). One image field was centered on an oblique dendrite and was photographed through 91 serial sections. The other image field was centered on an apical dendrite and photographed through 194 serial sections.

### Using Reconstruct to perform 3DEM analyses

Data files, calibration, image alignment, section thickness estimation, tracing, naming, 3D reconstruction, and quantitative analyses were generated using our custom designed software Reconstruct, which is freely available together with a detailed user’s manual (Data Citation 1)^35,59^.

#### Data files

The Reconstruct manual describes in detail the three types of data files. Briefly, these comprise: 1) The original image files. 2) The trace files with information about trace coordinates, colors, shapes, etc., as well as image alignment, contrast, and brightness parameters (seriesname.section_number). 3) One series file (seriesname.ser) contains information that is applicable across the serial sections, such as Z-traces (linear measurements that span multiple sections), the trace palette, calibration information, and any other information that is relevant to the entire series.

#### Contrast and brightness

We digitally optimize images for brightness and contrast in Reconstruct to visualize structures of interest while tracing. These changes are made to the trace files and do not permanently alter the original images; hence, these properties were adjusted appropriately during the identification and measurement of different structures (e.g., postsynaptic density, polyribosomes versus microtubules).

#### Image alignment

Standard protocols described in detail in the Reconstruct manual were used to align the images. Briefly, a pair of sections without artifacts (e.g., fold, tear, stain, or dust) was selected in the middle of the series. Beginning with these sections, fiducial traces were placed on cross-sectioned microtubules or mitochondria in the same location, and the linear alignment feature was applied to bring the images into alignment. One by one, sequential pairs were aligned up and down the series. The quality of the alignment was assessed by blending or flickering between the adjacent images. In a well-aligned series, the blended images appeared as a single well-focused image. If not properly aligned, the blended images appeared blurred or as double images in regions of sub-optimal alignment. Additional fiducial points were added in those regions to improve the alignment. If blended images could not be aligned adequately with the linear tool in Reconstruct, then the problematic section was skipped and its two adjacent sections were aligned instead. Usually, sections that could not be aligned had noticeable sectioning flaws (e.g., tears, folds, or compression artifacts). Deformal or quadratic alignment tools were used to bring each flawed section into alignment with its adjacent sections. These extreme alignments of flawed sections were not propagated throughout the series. Alignment coordinates were stored in the trace files; there were no alterations made to the original image files, as alignments are only applied when sections are viewed in Reconstruct. Copies of the aligned images were exported, with or without visualization of the traces, to generate figures. Aligned images could be similarly imported into other viewers and reconstruction programs. These strategies are documented in the Reconstruct manual.

#### Calibration

Pixel size was achieved relative to a diffraction grating replica (0.463 μm squares from Ernest F. Fullam, Latham, NY) that was photographed with each series. The Calibration function in Reconstruct was used to calculate the average pixel size across the image field resulting in a spatial resolution of ~2.2 nm/pixel. The original TIFF or BMP images are also available for image analysis or use in other programs (Data Citation 1)^56,60,61^.

#### Section thickness estimation

Section thickness was computed by dividing the diameters of longitudinally sectioned mitochondria by the number of sections they spanned^62^. In each aligned series, ten longitudinally sectioned mitochondria were identified and their diameters were measured (mitodia## is the name assigned for the diameter trace of each mitochondrion that was used). The width of the mitochondrion was measured at the same location on each section to determine the maximum (namely its diameter) and also to determine how many sections it spanned. The edge scores for the first and last sections of each mitochondrion were determined according to established criteria: 0.25 for light gray without visible cristae, 0.50 for medium gray without cristae, 0.75 for black without cristae, and 1 when mitochondrial cristae were visible^62^. For each mitochondrion, section thickness was estimated as the diameter divided by number of sections spanned; the low and high values were discarded, and the remaining values were averaged. Section thickness was calculated to be 49 nm for the oblique and apical dendrite volumes, which were photographed from the same serial sections. The section thickness of the spine volume (which was photographed from a different set of serial sections) was calculated to be 59 nm (Table 1 (available online only)).

#### Image volumes

The unaligned image volumes were subject to differential effects of sectioning and exposure to the beam in the electron microscope. Hence, the Reconstruct alignment features were used to bring objects into alignment relative to a central section that had no obvious artifacts. The aligned image domain areas were compared to the original unaligned image areas resulting in an average of ~10–15% adjustment to individual oblique or apical dendrite images across the series (Table 1 (available online only)). The spine volume was obtained from a series of EM negatives that had been digitally scanned and aligned using an older version of Reconstruct. Hence, the aligned spine image volume was not a relevant measure of image adjustment because the domains encompassed more than the image volume itself. We have rescanned and posted new unaligned images from the original spine volume negatives so that this image series can also be used to test auto-alignment and segmentation routines.

#### Densely reconstructed volumes

All of the dendrites, axons, and astroglial processes were traced in subdomains of each image volume to produce the densely reconstructed volumes (DR, Table 1 (available online only)). The spine volume occupied 42 sections for a total aligned volume of about 9.8 μm^3^. The oblique dendrite volume occupied 70 serial sections for a total aligned volume of 43.2 μm^3^. The apical dendrite volume occupied 101 serial sections for a total aligned volume of 178.2 μm^3^. Thus, in each case, substantial image volume surrounds the densely reconstructed volumes.

#### Tracing and naming

Table 2 (available online only) lists the trace name and type for each object in the DR volumes. The volume boundary markers were used to identify the borders of the same fields from section to section of the DR volumes. Each dendritic segment and unconnected spine fragment was given a unique number. Similarly, axons were given unique numbers with suffixes to identify their type (excitatory, inhibitory, or unidentified). Synapses were named according to their dendrite and axon numbers. The names of synapses on protrusions with more than one synapse (e.g., branched or multisynaptic spines) were further appended with ‘a’, ‘b’, ‘c’, etc. Traces made outside of the reconstructed volume were given the suffix ‘_o’ for ‘outside’. Subcellular objects were named according to the dendritic segment in which they were found, followed by object type and number. For example, the first mitochondrion identified in dendritic segment d008 was named d008mito01. Finally, glial processes were named according to their characteristics as astroglia (clear cytoplasm and glycogen granules), microglia (dark cytoplasm and lysosomes), and oligodendrocyte (forming myelin).

A stamp shape is one of the tracing tools that can be assigned a unique name and shape for ease of identifying objects or locations. The same shape can be assigned different names, such as d###p##, where d would be the dendrite and p a particular protrusion origin. The shape can be of the user’s choosing as described in the Reconstruct user’s manual.

Z-traces are traced across serial sections to compute the lengths of processes or objects in 3D^36^. They can be used, for example, to compute dendritic or axonal segment lengths. The Z-traces are routinely traced beginning from the starting section up and then from the last section down and then averaged, usually across 3–4 tracings. The Z-traces are stored in the .ser file and readily obtained for analysis when the series are loaded into Reconstruct.

#### Object and trace lists

Reconstruct provides output of object, trace, and other lists in a .csv format including quantitation of object volumes, surface areas, flat areas, counts, lengths, areas, perimeters, etc. that can be incorporated into spreadsheets for descriptive and statistical analyses. Each of these calculations is described in the user manual.

#### 3D reconstructions

Reconstruct also provides numerous approaches for display of 3D scenes. The user manual describes how to obtain the Boissonnat surfaces (used for 3D scenes in this report), boxes of specific dimension (used to set absolute dimensions of scale cubes in this report), and spheres (of particular sizes, used to represent polyribosomes in this report). Other styles, such as the traces, trace slabs, and cylinders, are also available.

### Visualization in the Open Connectome Project

#### Data ingest and storage management

EM images and annotations have been exported from Reconstruct and ingested into the OCP spatial databases (i.e., databases that support queries in 3D space). Data were exported by reading files with the MATLAB scripts for reading Reconstruct projects (http://GitHub.com/openConnectome/ReconstructImport), converting annotation contours into dense spatial arrays, merging annotations and grouping them by class (axon, dendrites, glia, and synapses), and outputting image files for EM and each group of annotations. Ingest scripts in Python assembled multiple image files into a 3D volume that is posted to OCP Web-services. Once ingested, OCP built a multi-resolution image hierarchy that recursively scaled data by a factor of two in the imaging plane (x and y dimensions) at each lower resolution. EM data and each annotation group defined an OCP *project* that can be queried, downloaded, or visualized independently. All projects have been co-registered within an OCP *dataset* that describes a common spatial domain.

#### Web visualization with CATMAID

Spatial databases in OCP can be visualized through a web browser using the Collaborative Annotation Toolkit for Massive Amounts of Image Data (CATMAID, http://catmaid.org) as developed by Saalfeld *et al.* ^63^ CATMAID represents 3-dimensional data as consecutive images, presenting one image (in the original imaging plane) at a time within the browser. The interface includes pan and zoom controls that navigate data within a resolution hierarchy. CATMAID includes an overlay capability to place layers of annotations on top of EM data and controls to adjust transparency of layers.

#### Data delivery and caching for visualization

CATMAID requests data from OCP as tiles, typically 512 by 512 pixels, at a given resolution. OCP provides Web-services for individual tile requests that extract image planes from volume databases. To accelerate visualization when accessing nearby slices or when viewing data repeatedly, OCP manages a hierarchy of caches. First, an in-memory tile cache is maintained in the OCP data cluster using *memcached* (http://memcached.org/). The cache contains the most frequently accessed tiles, subject to the cluster’s memory capacity. Tile requests that miss the memory cache are rendered on demand by the Web server. When serving such a cache miss, OCP must read a volume of data around the requested slice, because data are stored in 3D cuboids. OCP takes the entire volume of the data, renders it into slices, and stores it on a disk cache, called the OCP tile cache (available at https://github.com/openconnectome/ocptilecache). We loaded the disk cache using an asynchronous background process. This approach populates the cache without slowing down the user interface. Requests to nearby regions of data fetch the data from the disk cache (e.g., when scrolling through serial sections at the same location). This avoids redundant I/O and computation; data are read once and rendered once. The disk cache is deployed independently from the volume databases. We deploy OCPtilecache on independent hardware to reduce load on the database cluster. The cache can also be deployed locally to clients so that data can be fetched over low-latency networks. When configured in this way, OCPtilecache serves as a content distribution network.

Data are publicly available through OCP RESTful Web services for download and data-intensive analysis^29^ and interoperate with OCP tools, such as computer vision pipelines (CAJAL, https://github.com/openconnectome/ocptilecache) and Web visualization, such as CATMAID, as indicated above.

#### Data delivery

Web services can be accessed programmatically within user-facing analysis tools, such as Python, Matlab, and R, which allows scientists to access data and metadata interactively through their preferred analytics platform. OCP Web services implement a wide variety of data-intensive queries that compute spatial and statistical analysis on big data on the server cluster and return smaller query answers that have been processed, filtered, or otherwise enriched. In this way, OCP implements a platform that supports exploratory data analysis on petascale data over the internet.

### Code availability

Currently, there are three approaches to obtain access to the Reconstruct alignment and trace files (Data Citation 1, Tools) including Reconstruct, Fiji/TrakEM2, and OCP. Here we describe briefly how to obtain access to useful code for manipulating these data in the future.

#### Reconstruct

The immediate approach is to download the Reconstruct software directly and follow instructions on how to load the images and the trace and series files and use it as described in the manual and above. The most reliable version of the Reconstruct program is the 32-bit Version 1.1.0.1 running on a Microsoft Windows platform. The executable for the Reconstruct software and user manual and detailed instructions for how to access all of the data files and related software are available at OCP (Data Citation 1, Tools). It takes a new user minutes to begin using Reconstruct for viewing and interacting with the existing data following the ‘Quick Start’ instructions in chapter 1 of the manual. After about 1–2 h, the new user can become sufficiently proficient to begin adding new traces and to visualize objects in 3D. Obviously, to become expert in using all of the tools that exist in Reconstruct requires a more sustained effort. There is a worldwide user group, and numerous labs have downloaded and published using this software (see for examples http://synapseweb.clm.utexas.edu/).

Reconstruct data were ingested into the OCP for browser display, as discussed above and further in this paper. MATLAB scripts are available to read the Reconstruct project files (http://GitHub.com/openConnectome/ReconstructImport) and can be found with the link (Data Citation 1). In addition, efforts are under way to upgrade Reconstruct for 64-bit machines. The 32-bit source code is available by request. The original raw images and Reconstruct trace files are deposited for future exploration and analyses (Data Citation 1).

#### Reconstruct to Fiji/TrakEM2

Fiji/TrakEM2 includes a plugin that reads in and displays a Reconstruct project (this plugin http://fiji.sc/Reconstruct_Reader is automatically loaded into current versions of Fiji). To load a Reconstruct project, first download and extract the relevant project files (http://openconnecto.me/harrisdata/). Open Fiji and use the File menu dropdown to select ‘Import -> Read Reconstruct Project’. Browse to and select the .ser file for the project you want to open. Fiji will load the Reconstruct project and display it using TrakEM2 in Fiji. Currently TrakEM2 does not support application of nonlinear alignments, hence if deformal or quadratic alignments were used on some sections in Reconstruct, those would need to be re-aligned in TrakEM2, which is true for some sections in the apical and oblique volume sets. Since Reconstruct projects store images and annotations separately from the alignment transformations, contours in each section remain properly aligned with the underlying image.

#### Reconstruct to Open Connectome

Open Connectome has made available several Matlab scripts on GitHub (https://github.com/openconnectome/ReconstructImport). The scripts convert Reconstruct projects to file formats suitable for upload to Open Connectome. A Reconstruct Project consists of a set of raw images and corresponding XML files, each of which contains alignment and contrast transformations together with contour annotations for that image. First, the transformations must be applied to the raw image files. In addition, contours in the XML files must be converted to PNG files where each pixel corresponds to a number that is an unique identifier for each contour. Additionally, the scripts can merge annotation images for faster upload to Open Connectome’s database. All code is documented in the README.md file, which is published along with the Reconstruct import code on GitHub.

#### Open Connectome data download

In addition to web visualization, Open Connectome supports data download directly from web URLs. Instructions for data download, including supported URL calls, are available (Data Citation 1, Tools). Open Connectome also provides CAJAL, a software package that allows Matlab users to download and interact with Open Connectome datasets. The latest version of CAJAL is available on GitHub (https://github.com/openconnectome/CAJAL). An example script for downloading and visualizing the data is available (Data Citation 1, Tools).

## Data Records

### Dense reconstructions from Reconstruct projects

**Figure 2:**
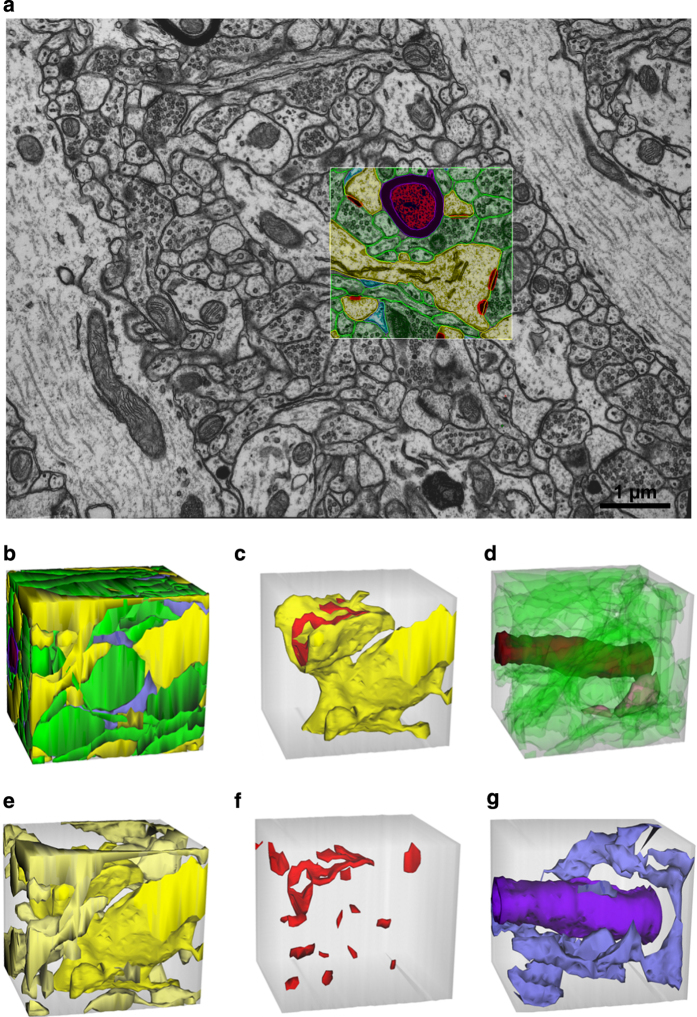
EM and 3D reconstructions from the volume that surrounds a large dendritic spine with the synapse (named d01c01Ax54e). (**a**) Sample EM illustrating a single section plane with spiny dendrites (yellow), excitatory axons (green), synapses (red), astroglial processes (light blue), and a myelinated axon (red center axon surrounded by purple sheath). (**b**) 3D reconstructions of all objects traced in the spine volume. (**c**) 3D of a dendritic spine protruding from the portion of the dendritic shaft (yellow) contained in this volume. The synapse on the head of this large spine has an irregularly shaped, perforated PSD (red). (**d**) 3Ds of all unmyelinated excitatory axons (green), an unmyelinated inhibitory axon (pink), and a myelinated axon (dark red) that entered this volume. (**e**) 3Ds of all dendritic segments (yellow) and (**f**) all excitatory synapses in the volume. (**g**) 3Ds of an astroglial process (light blue) and myelin (purple). The square outlined in (**a**) and the gray cubes in (**b**–**g**) are 2 μm per side, though the orientation foreshortens some of these edges in the 2D plane of this figure.

**Figure 3:**
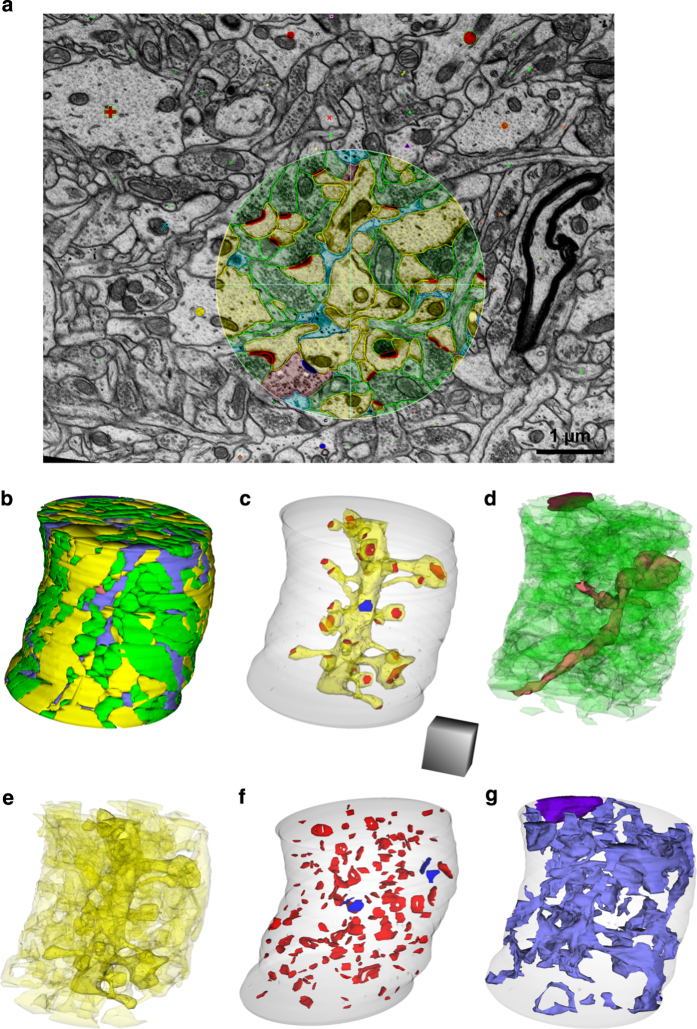
EM and 3D reconstructions from the volume that surrounds an oblique dendritic branch (named d03). (**a**) Sample EM illustrating a single section plane with spiny dendrites (yellow), excitatory axons (green), an inhibitory axon (pink), synapses (red), and astroglia (light blue). (**b**) 3D reconstruction of all objects traced in the oblique dendrite volume. (**c**) 3D of the oblique dendrite (d03, yellow) encased in the surrounding translucent cylindrical volume, its excitatory (red) and one inhibitory (blue) synapses. (**d**) 3Ds of all unmyelinated excitatory axons (green), unmyelinated inhibitory axons (pink), and a myelinated axon (purple) that entered this volume. (**e**) 3Ds of all spiny dendritic segments (yellow). (**f**) All excitatory (red) and inhibitory (blue) synapses in the volume. (**g**) 3D of all the astroglial processes (light blue) and a myelin sheath (purple). Cylinder is 4 microns in diameter and scale cube is 1 μm^3^.

**Figure 4:**
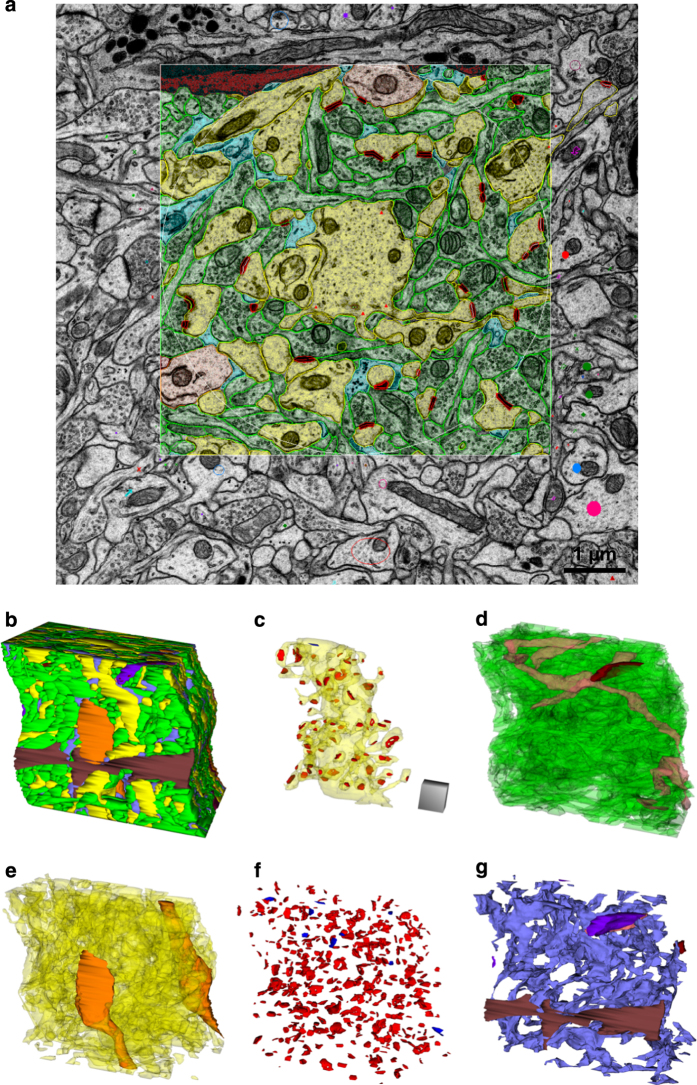
EM and 3D reconstructions from the volume that surrounds an apical dendrite (named d000A). (**a**) Sample EM illustrating a single section plane through the apical dendrite volume with spiny dendrites (yellow), nonspiny dendrites (orange), excitatory axons (green), excitatory synapses (red), astroglia (light blue), microglia (dark brown), and colored stamp shapes identifying objects that entered the volume on other sections. (**b**) 3D reconstruction of all objects traced in the apical dendrite volume. Large microglial process (dark brown) and interneuron dendrite (orange) face outward. (**c**) 3D of the central apical dendrite (yellow, translucent) with all of its excitatory synapses (red) and its one inhibitory shaft synapse (blue). (**d**) 3Ds of all unmyelinated excitatory axons (green), unmyelinated inhibitory axons (pink), and the edge of a myelinated axon (purple) that entered this volume. (**e**) 3Ds of all spiny (yellow) and non-spiny (orange) dendritic segments. (**f**) All excitatory (red) and inhibitory (blue) synapses. (**g**) All astroglial processes (light blue), a microglial process (dark brown), and a myelinated axon (purple). Scale cube is 1 μm^3^.

Three densely reconstructed regions (Fig. 1b) surrounded a dendritic spine (Fig. 2), an oblique dendrite (Fig. 3), and an apical dendrite (Fig. 4). For illustrative purposes, the traces were superimposed on a central EM image from each of the respective series (Figs 2,3,4a), followed by all of the reconstructed objects (Figs 2,3,4b) and the central spine or dendrite shown with its excitatory and inhibitory synapses (Figs 2,3,4c). The excitatory axons, inhibitory axons, and portions of a myelinated axon were reconstructed in 3DEM (Figs 2,3,4d). All of the other spiny dendrites were illustrated in 3DEM together (Figs 2,3,4e). There were only two nonspiny dendrites, which were presumably from interneurons, and both entered the volume surrounding the apical dendrite (Fig. 4e). Reconstruction of all of the excitatory and inhibitory synapses illustrated their distribution throughout the volumes (Figs 2,3,4f). Finally, the astroglia, myelin, and microglial processes were shown in 3DEM (Figs 2,3,4g). The overlap between astrocytic domains in the hippocampus occurs only at the borders of their processes, thus it is unlikely that the reconstructed astroglial processes in each volume arose from more than one astrocyte^64,65^. The number and identities of objects in each densely reconstructed volume are summarized in Table 2 (available online only) and in the figures described below.

The spine volume encompassed a portion of a large dendritic spine emerging from a small dendrite that traversed neuropil located between two longitudinally sectioned dendrites (Fig. 2a). The densely reconstructed volume formed a cube (Fig. 2b). The large dendritic spine had a perforated synapse and occupied most of the central region of this volume (Fig. 2c). A small myelinated axon ran perpendicular to the image plane and was visualized amongst 18 unmyelinated excitatory axons, one inhibitory axon, and 38 axon portions that were too small to identify unequivocally (Fig. 2d). Portions of eight other dendrites or spines entered the cube (Fig. 2e). These processes formed 17 excitatory but no inhibitory synapses in this volume (Fig. 2f). The one myelin sheath and all of the astroglial processes were reconstructed (Fig. 2g).

The densely reconstructed oblique dendrite volume formed a cylinder (Fig. 3a,b) around the central dendrite (Fig. 3c). The volume contained 97 excitatory, 3 inhibitory, and 59 unidentified axons (Fig. 3d) and 20 dendritic segments (Fig. 3e). There were 165 excitatory synapses and 4 inhibitory synapses in the volume (Fig. 3f). In addition to the astroglial processes, the edge of a myelinated axon skimmed the cylindrical volume (Fig. 3g).

The apical dendrite was presumed to be apical due to its large size relative to the surrounding oblique dendrites (Fig. 4a). The densely reconstructed volume created an irregular rectangle centered on the apical dendrite as it coursed through the neuropil (Fig. 4b,c). This volume contained 281 excitatory, 8 inhibitory, and 114 unidentified axons (Fig. 4d). There were 40 spiny dendrites and 2 nonspiny, presumably inhibitory dendrites that entered the volume (Fig. 4e). These processes formed 488 excitatory synapses and 13 inhibitory synapses within or at the edge of the densely reconstructed volume (Fig. 4f). In addition to the extensive astroglial processes, part of a microglia cell entered the volume along with part of a myelinated axon (Fig. 4g).

### Excitatory axon trajectories

**Figure 5:**
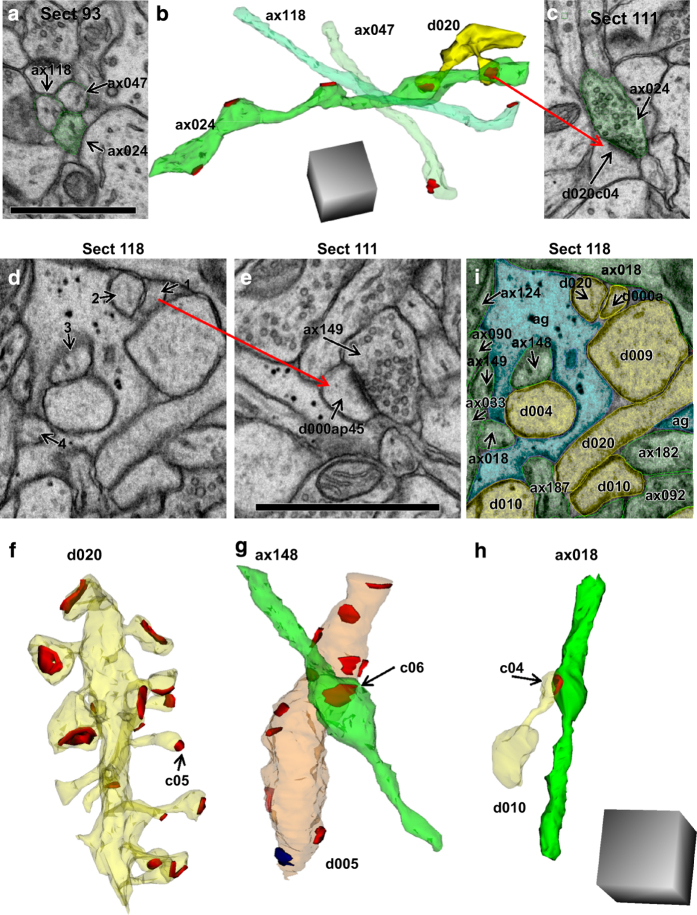
Trajectories of excitatory axons in the apical dendrite volume illustrate necessity of 3DEM for unambiguous identification. (**a**) Three axonal profiles appeared to be identical on one section. (**b**) 3D reconstruction revealed the diversity in synaptic contacts along their length. Five synapses were made along ax024 (7.9 μm) in this volume while the other two (ax118, 5.3 μm and ax047, 6.0 μm) made only one synapse each. These axons appear to run in parallel in section 93; however, 3DEM shows instead that their paths crossed. The illustrated dendrite (d020, yellow) had two spines that synapsed (red) on different varicosities of the same axon (ax024). (**c**) EM illustrating one of the synapses between ax024 and d020 (named d020c04Ax024e). (**d**) EM with 4 ambiguous objects that look essentially identical on this section. (**e**) Object 1 was identified as a dendritic spine neck, as its head formed an asymmetric synapse with ax149 on section 111. (**f**) Object 2 from section 118 was similarly discovered to be part of a spine on dendrite d020 (synapse named d020c05Ax046e). (**g**) Object 3 from section 118 was found to be part of ax148, which made one synapse in the reconstructed volume with a nonspiny dendritic segment (d005, orange) that had 12 asymmetric, presumably excitatory synapses (red) and one symmetric, presumably inhibitory synapse (blue) along its length. (**h**) Object 4 was identified as ax018, which formed one synapse (d010c16Ax018e) in the reconstructed volume with dendrite 10 (d010). (**i**) All of the objects on section 118 were identified through 3D reconstructions and are colorized and labeled in this EM (spiny dendrites, yellow; excitatory axons, green; glia, light blue). All scale bars are 1 micron, and scale cubes are 1 μm^3^.

3DEM was necessary to distinguish axons, dendrites, spines, and glial processes (Fig. 5). For example, section 93 from the apical series revealed three axons that were close together and were nearly identical in appearance (Fig. 5a). These small axonal processes were distinguished from cross-sectioned spine necks by reconstructing them across serial sections, which revealed varicosities containing presynaptic vesicles and synapses along their lengths (Fig. 5b,c). While these three axons appeared to run parallel to one another on section 93, they in fact ran perpendicular to one another in 3DEM (Fig. 5a). Axon 47 and axon 118 were nearly uniform along their lengths and each made only one excitatory synapse within this volume (Fig. 5b). In contrast, axon 24 had multiple varicosities of different sizes and made five excitatory synapses within the volume, two with spines from the same dendrite (Fig. 5b,c).

To illustrate further the requirement for 3DEM, Fig. 5d shows a portion of section 118, on which four neural processes appear strikingly similar to the axons on section 93. However, when process 1 was followed through seven adjacent sections, it was found to be a dendritic spine synapsing with axon 149 on section 111 (Fig. 5e). Process 2 was discovered to be part of dendritic spine number 5 on dendrite 20 (Fig. 5f). In contrast, process 3 was discovered to be axon 148 that synapsed on the shaft of dendrite 5 (Fig. 5g), and process 4 was discovered to be an axon synapsing with a spine on dendrite 10 (Fig. 5h). Due to the homogeneous appearance of these processes on individual sections, accurate identification would have been impossible in a single section analysis. Hence, 3DEM allowed for the unambiguous identification of each process on section 118 (Fig. 5i).

### Calculation and 3D visualization of synapse areas

**Figure 6:**
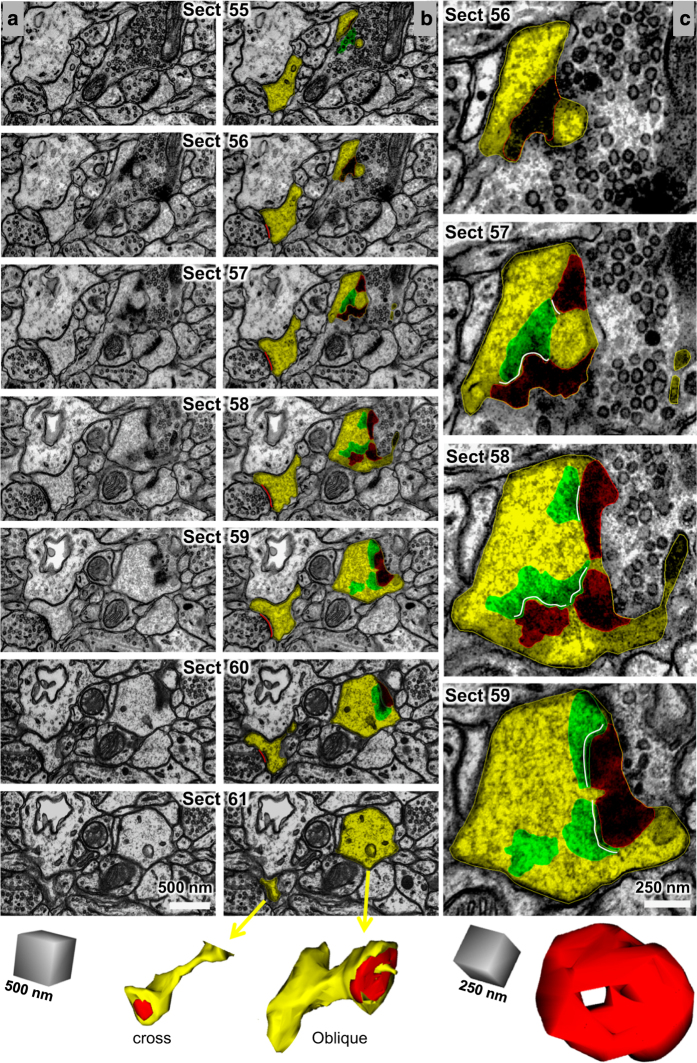
Standard strategies for measuring and visualizing asymmetric synapses. Column (**a**) shows serial images through two dendritic spines were exported from Reconstruct with the contrast enhanced for the PSD, hence these images appear somewhat darker than those that were optimized for grayscale in other figures. Column (**b**) shows the same serial images as in (**a**) exported this time with spine and PSD traces illustrated. Upon reconstruction, the cross-sectioned synapse (named d01c01Ax59e_o) had a continuous, macular shape, while the obliquely sectioned synapse (named d07c01Ax17e_o) had a perforation with a spinule emerging from the spine head. Column (**c**) A subset of sections through the obliquely sectioned synapse shown at higher magnification to illustrate how the synapse was measured and the location of the spinule (sp). The reconstruction of the synapse rotated to visualize the perforation from the top of the spine head. Scale cubes are labeled for the lengths along one side.

Asymmetric excitatory synapses were identified by the postsynaptic density (PSD), which has a protein-rich content that is darkly stained by uranyl acetate and lead citrate. Synapse areas were traced as illustrated in Fig. 6. Synapses were first categorized by their orientation with respect to the cutting plane. Cross-sectioned synapses exhibited distinct pre- and postsynaptic plasma membranes, a synaptic cleft, and presynaptic vesicles (Fig. 6, left spine). En face PSDs occur when the section is cut exactly parallel to the entire synapse such that the entire ‘face’ of the synapse is visible. Oblique PSDs occur when the section is cut at an angle such that the PSD slopes across more than one section (Fig. 6, right spine). Synapses with complex, irregular shapes can have a combination of these orientations. Because presynaptic vesicles contained within a section can be obscured by the PSD of en face or obliquely sectioned synapses, vesicle counts are less reliable for synapses sectioned at these orientations. Synapses with irregular shapes were often sectioned at a combination of all three orientations with respect to the cutting plane.

Here we briefly describe how we obtain standard calculations of synaptic areas. Additional information is provided in the user manual. Synaptic areas were calculated based on the extent of the apposition between the pre- and postsynaptic structures along the PSD. Because these surfaces are irregular, Reconstruct provides a means to calculate this apposition, or any similar type of apposition, which is called the flat area. This flat area calculation is distinguished from standard surface area calculations, which are instead based on the perimeters of enclosed volumes. Synapse flat areas were named as indicated in Table 2 (available online only) and computed by the following formula, where an open contour is a line and a closed contour encompasses an area:

$$\mathrm{Synapse}\;\mathrm{flat}\;\mathrm{area}=\Sigma_{\mathrm{sections}}[\Sigma_{\mathrm{open}\;\mathrm{contours}}\mathrm{length}\times \mathrm{section}\;\mathrm{thickness}+\Sigma_{\mathrm{closed}\;\mathrm{contours}}\mathrm{area}].$$

For portions of the synapse that were cross-sectioned, an open contour was placed along the length of the PSD on each section. For en face synapses, a closed contour was placed to encircle the dark area of the PSD and measure its area. Since the PSD has depth within the cytoplasm, it was necessary to determine what portion of an obliquely sectioned synapse reflected that thickness, and what portion was the synapse flat area. To determine whether a PSD was actually obliquely sectioned, its width where it overlapped the presynaptic axon was measured. If this width was greater than or equal to twice the width of a neighboring, perfectly cross-sectioned PSD, then a closed contour was placed around the portion of the obliquely sectioned PSD, which overlapped with the adjacent axon. In this way, the depth of the PSD thickness was not included in the flat area. Then these closed areas were copied to adjacent sections (green) to serve as guides to determine where an open contour (white) should be placed to measure the portion of an obliquely sectioned PSD that traversed adjacent sections (Fig. 6, starting at section 56, right column). For training purposes, the synapse flat areas were traced along the central dendrites of each volume: D01 in the spine volume, D03 in the oblique dendrite volume, and D000A and D000B in the apical dendrite volume. Synapse flat areas were also traced and quantified for five additional dendrites (D001, D002, D007, D009, and D010) in the apical dendrite volume.

To visualize 3D surfaces using the Boissonnat algorithm^66^, as implemented in Reconstruct, the contours must be closed and span at least 2 sections. Hence, to visualize synapse areas, closed contours were created to encapsulate the PSD and part of the presynaptic active zone for all cross-sectioned portions of synapses (Fig. 6, bottom left). For obliquely sectioned synapses, the closed contours were copied and renamed (Fig. 6, bottom right, notice that the perforation was maintained). Finally, if a synapse was sectioned perfectly en face, the same closed contour was copied on the next section on the presynaptic side of the synapse to create the necessary depth for 3D visualization. For illustrative and training purposes, all of the excitatory (asymmetric) and inhibitory (symmetric) synapses were traced for 3D visualization in each of the three densely reconstructed volumes.

### Other structures identified or traced in Reconstruct for training purposes

For training purposes, a Z-trace was created to measure dendritic lengths by placing it in the center of the dendritic shaft across serial sections of five dendrites in the apical dendrite volume (D001, D002, D007, D009, and D010). Typically, we hold the trace in one location until the dendrite trace moves sufficiently such that the center of the Z-trace is outside the dendrite, then we re-center the trace. This avoids over-tracing down the center of the dendrite (additional instruction is in the manual). These traces can be accessed by loading the project into Reconstruct and clicking on Objects/Z-traces. When the list comes up, double-clicking a Z-trace will place it into the 3D viewing window. In this way, a user can visualize a length trace within a translucent object, such as a dendrite. Stamp shapes were positioned at the spine origins and shaft synapses along these dendrites. Spine head diameters were traced at their widest diameter, either on a single section or by using a Z-trace tool, for the completed spines on each of these five dendrites.

**Figure 7:**
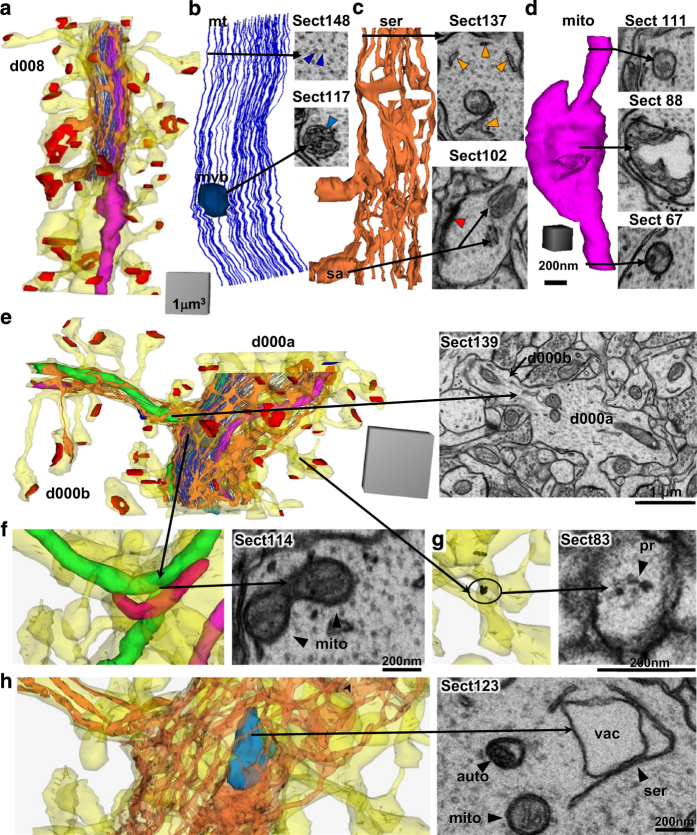
3Ds and representative EMs of subcellular structures. (**a**–**d**) Dendritic segment d008 from the apical dendrite volume. (**a**) 3D reconstruction of the dendrite (yellow), excitatory synapses (red), and a subset of organelles that were traced and reconstructed on sections 100–153. (**b**) Enlarged view of microtubules (mt, royal blue lines) and EM where they appear circular and regularly spaced throughout the shaft of the dendrite on section 148 (royal blue arrowheads). A circular stamp shape was placed manually in the center of each microtubule. Since stamp shapes are closed objects they can represent objects in 3D. The jagged appearance is due to subtle misalignments of the image sections and manual placement of the stamps. 3D of a multivesicular body (teal ovoid) and EM as it appears (teal arrowhead) on section 117. (**c**) 3D of SER (orange) and EM from section 137, where the SER was tubular and distributed throughout the dendritic shaft. A spine apparatus (sa, arrow), consisting of folds of SER interspersed with dense plates, is illustrated from section 102 in a spine head where part of the PSD was also present (psd, red arrowhead). (**d**) 3DEM of the mitochondrion (mito, fuchsia) where it appears compact on sections 67 and 111, but distended between where it might be dividing or in a different functional state as visualized on section 88. Scale cube in (**d**) is 200 nm per side and scale bar is 200 nm for all 3Ds and EMs, respectively, in (**b**–**d**). (**e**–**h**) 3Ds and representative EMs of subcellular structures in the branched central dendrite from the apical dendrite volume. (**e**) 3D reconstruction of the dendrite (yellow), excitatory synapses (red), and a subset of organelles that were traced and reconstructed on sections 100–152. EM from section 139 shows d000B branching off from d000A. (**f**) 3DEM of 3 mitochondria (green, pink, fuchsia) and polyribosomes (black spheres). An EM from section 114 shows the branch point of the green mitochondrion (mito, arrowheads) as it enters d000B and also continues along the length of d000A. (**g**) 3D of a portion of the dendrite where spine contains a polyribosome. Section 83 illustrates an EM from section 83 of a polyribosome in a spine neck protruding from dendritic segment d000A (pr, arrowhead, named d000Arn20 in the trace files for Reconstruct). (**h**) 3DEM of SER encircling a vacuole (teal blue) of an autophagosome complex. EM from section 123 shows the vacuole (vac) encircled by SER (ser, arrowhead) in close proximity to an autophagosome (auto, arrowhead) and a mitochondrion (mito, arrowhead). Scale cube 1 μm^3^ for 3DEM in (**e**) and 0.125 μm^3^ (500 nm per side) for 3DEMs in (**f**–**h**) and scale bars are 200 nm in (**f**–**h**).

Also for training and identification purposes, subcellular structures were named (Table 3 (available online only)) and traced in dendrites D008 and D000A-B from the apical dendrite volume (Fig. 7). The short reconstructed dendritic segments (Fig. 7a) contained microtubules that typically spanned their entire length (Fig. 7b), as would be expected from our prior work showing them to be about 80 μm long in the CA1 dendrites^67^. SER formed a continuous network of tubules, branches, and lakes in the dendritic shaft (Fig. 7c)^52^ and occasionally expanded into dendritic spines to form a spine apparatus (Figs 2a, 7c). Mitochondria spanned the full lengths of these dendritic segments. One appears to have been in multiple functional states, or dividing, with both compact and distended regions (Fig. 7d). The apical dendrite (Fig. 7e) had a mitochondrion that branched into a side dendritic branch (Fig. 7f). Polyribosomes occurred in the dendritic shaft and some spines (Fig. 7g). Endosomal compartments (Table 3 (available online only)), including autophagosomes (Fig. 7h), were also identified and traced in these two dendrites. Multivesicular bodies (Fig. 7b) were included in the endosomal compartments, although some might in fact be exosomes^68^. Standard names for all of the traced subcellular components in Reconstruct are summarized in Table 3 (available online only) along with the total number of objects for each component. The OCP sliders are described next.

### Web visualization with CATMAID in the Open Connectome Project

All three volumes are available online for visualization in a web browser. Links to visualized data are provided on the landing page of the OCP for SynapseWeb (See Fig. 1a, above, Data Citation 1). The Visualize button takes the viewer to a CATMAID landing page for all available SynapseWeb datasets, whereas the red buttons under Visualize take the viewer directly to the individual dataset pages. While any OCP data can be visualized with CATMAID, we have selected and grouped individual objects (Table 1 (available online only)) into four subcategories (axons, dendrites, synapses, and glia) from the full datasets to facilitate fast loading times in the web interface (Table 4 (available online only), with total number of objects in each category). To visualize and interact with specific objects, such as synapse 1 on dendrite 1, the specific image data and related Reconstruct files can be obtained under Download. To obtain free software that can be used to quantify or interact with specific objects, use the Tools links to obtain software and instructions on how to download and use the software.

**Figure 8:**
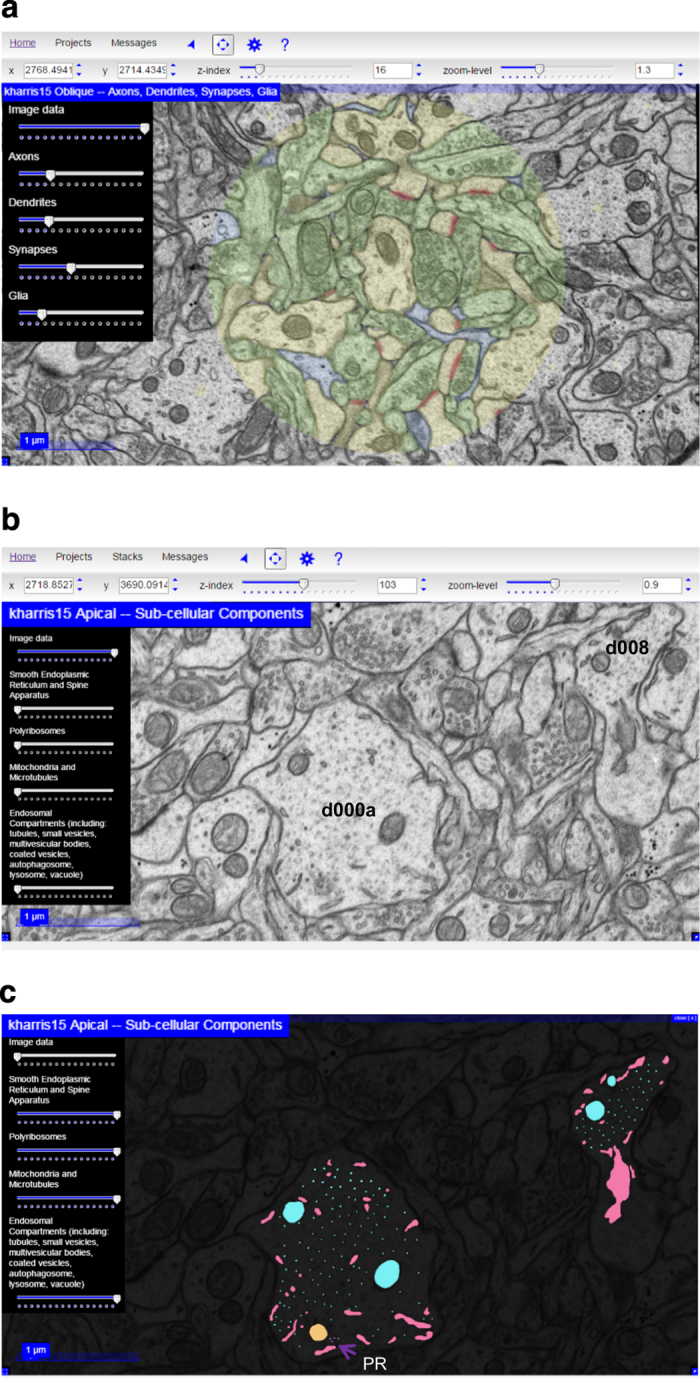
Subcellular components in two dendrites in the apical dendrite volume visualized at OCP. (**a**) Web browser visualization of the apical volume using CATMAID with opacity controls for the Image data set at 100%. Each of the layers—Axon (green), Dendrite (yellow), Synapses (red), and Glia (blue)—are adjusted for translucent overlay on the Image. Controls for z-index (section number) and zoom-level (see text) are also available in the top menu bar. (**b**) Image data at 100% to illustrate d000A and d008, all other layers set to zero. (**c**) Image data at nearly 0% and all other sliders are set to maximum to illustrate various groups of subcellular components, including smooth endoplasmic reticulum (pink), polyribosomes (purple, PR—arrow), mitochondria and microtubules (turquoise), and endosomal compartments (peach).

CATMAID enables interactive visualization through opacity, z-distance, zoom, and pan controls (Fig. 8a). Each slider on the left hand side controls the *opacity* of a layer of the 2D image where the first layer is the EM image and then each group of annotations is layered on top. Sliders control the opacity of annotation layers and allow users to hide or reveal the image or annotation layers completely, or as translucent overlays. By default, the EM Image slider is set to 100% opacity and the annotation layer sliders are set to 25% opacity. The menu bars at the top allow the user to manipulate position within the image stack (z-distance) and to zoom into or out of the current view. Each page through the *z-distance* corresponds to one section thickness. The z-index slider allows the user to jump to an arbitrary z-index (section) or click through z-indices (sections) sequentially with the up and down arrows to the right of the slider. Navigation through z-indices is also possible with the scroll wheel of a mouse or trackpad of a laptop computer. Users can also manually enter a z-index in the textbox between the slider and the arrows. *Zoom* level controls are located to the right of the z-index controls and operate identically. For these data sets, the image files and annotations have been scaled to three lower resolutions. Specific image sizes for each resolution are available at (Data Citation 1: http://w.ocp.me/datum:harris15#resolution_hierarchy). Integer zoom levels are native resolutions from the OCP database, and decimal zoom levels are a magnification of the image. In this way, as the user progresses to smaller zoom numbers, the resolution and magnification increase to allow objects to be scrutinized. Users can *pan* to different *XY locations* by adjusting the value in the X and Y box at the far left or by clicking the left mouse button and dragging the image.

The blue star to the left of the ‘?’ in the top menu-bar opens an additional settings menu where users can enable or disable a grid overlay and adjust its parameters. Reference lines in the x- and y-directions are also available by checking the Display Reference Lines checkbox in the menu bar. The links at the very top of the screen allow users to jump quickly between projects. Hovering with the mouse over the Home link will bring up a menu listing of all available datasets, grouped by research group or paper. Hovering over the Projects menu brings up all datasets available to a particular group or paper.

The subset of dendrites with reconstructed subcellular compartments can be observed in OCP by clicking first on Visualize in the landing page (Fig. 1a, above) and then on kharris15 Apical—Sub-cellular components (Fig. 8b). The dataset for the subcellular components contains four sliders that adjust the opacity differentially for smooth endoplasmic reticulum, polyribosomes, mitochondria and microtubules, and endosomal compartments (Fig. 8c).

## Technical Validation

Release of these densely reconstructed volumes of hippocampal neuropil is intended to serve multiple purposes. They provide accurate identification of axonal, dendritic, and glial processes as well as example subcellular dendritic structures to train students and researchers who are new to ultrastructural analysis. We provide detailed instruction to standardize reconstruction that estimates synaptic areas cut in all orientations. Additional images of the neuropil surround the reconstructed volumes so that once new users recognize the reconstructed objects, the surrounding images can be used to test that knowledge. The original images are provided at high resolution without superimposed alignment scaling, so that alternative approaches can be tested.

For all of our prior publications, analyses have been done blind as to experimental conditions to control for potential biases. For these three densely reconstructed volumes, all of the axons, dendrites, and glial processes were followed individually across serial sections and their outlines were traced and named on each section. After the initial tracing, in a process known as curation, all dendrites, axons, synapses, and organelles were re-examined to assess identification accuracy^69^. To achieve unambiguous identification, some of the processes were followed outside the dense reconstructions and stamp shapes with the specific object names placed along their lengths. Where viewers disagreed initially, discussions resulted in consensus. To the best of our knowledge, these tracings are accurately and completely identified within the densely reconstructed sub-volumes. The boundaries were manually traced and in some cases may overlap or stray slightly from the boundaries of the plasma membranes, especially where objects are obliquely sectioned.

Extracellular space (ECS) was thoroughly investigated in the apical dendrite volume in a prior publication and hence was not addressed here^52^. New tools embedded in VolRover were used to remove all of the trace overlaps for that analysis of extracellular space^52,70^. The tools in VolRover have not yet been integrated with Reconstruct or OCP; therefore, other programs are needed to explore ECS. It is worth noting, as discussed in our prior publication, that even with the VolRover improvements 3DEM likely underestimates the ECS because accurate determination of membrane boundaries can be especially difficult where membranes are obliquely sectioned. In the oblique planes from these ~50–60 nm sections, overlying membranes obscure some of the ECS as it would be buried within the depth of the overlapping objects in a section. ECS can be further reduced by the chemical fixation, which causes astroglial processes to swell relative to cryo-electron microscopy (G. Knott, personal communication), and possibly the dehydration steps used for ultrastructural analyses^52,71^. The degree to which ECS was lost versus obscured by overlying oblique membranes is an open question whose answer will require future 3D tomographic reconstructions, which provide z resolutions on virtual image sections in the range of 2–3 nm (e.g., http://bio3d.colorado.edu; RRID: nif-0000-31686)^51^.

We estimated that the alignments obtained in Reconstruct starting at a central section with no obvious artifacts required on average ~10–15% overall scaling of the images for the apical and oblique dendrite volumes (Table 1 (available online only)). The effect was greatest at the edges of the images^58^, and hence the densely reconstructed volumes were contained near the center of the image volumes. New tools that elastically align simultaneously over multiple sections might serve to distribute the adjustments for image registration more uniformly across the series, although the impact on the values determined from Reconstruct when objects remain near the image center should be minimal^55,56,60^.

These dense reconstructions provide ground truth identifications for all of the dendrites, axons, glia, and synapses, together with high quality reconstructions of representative subcellular structures. Thus, they provide a crucial basis from which to generate and test the validity of computer segmentation algorithms, to reduce operator involvement, and to provide accurate densely reconstructed volumes more quickly. We posit that these high-resolution images will serve to develop machine learning algorithms that can automatically identify structurally distinct objects, such as the spine apparatus, that are highly important for local synapse function but occur infrequently in the neuropil. We hope that new tools will also help to identify locations of ambiguity on the images and help to improve the reliability of reconstructions and surface measurements and renderings. Such automated discovery tools should greatly facilitate future experiments, which will elucidate how specific structures become altered during normal processes (such as learning) or in disease states (such as Alzheimer’s or epilepsy) and then provide new and more specific targets for treatment.

## Additional Information

Tables 1,2,3,4 are only available in the online version of this paper.

**How to cite this article:** Harris, K. M. *et al.* A resource from 3D electron microscopy of hippocampal neuropil for user training and tool development. *Sci. Data* 2:150046 doi: 10.1038/sdata.2015.46 (2015).

## Supplementary Material



## Figures and Tables

**Table 1:** Image and 3DEM volumes

**Volume Name**	**Image file and Domain names**	**Image sections**	**Section thickness**	**Unaligned Image Volumes**	**Aligned Image Vol**	**Absolute Alignment Effects**	**DR Sections**	**Aligned DR Volume**
**Spine**	k24	60	59 nm	169.5	NA	NA	42	9.8 μm^3^
**Oblique dendrite**	R34CA1-B_S12	91	49 nm	357.0	323.1	9.7+/−4.5%	70	43.2 μm^3^
**Apical dendrite**	R34CA1-B_S12	194	49 nm	783.1	865.8	14.4+/−12.6%	101	178.2 μm^3^
All of the images were from the middle of stratum radiatum in area CA1 of a 77 day old male Rat of the Long-Evans strain that had been perfusion fixed *in vivo* as described in the methods. The images for the oblique and apical dendrite volumes were obtained from different parts of one set of serial sections using a transmission electron microscope with a digital camera as discussed in the methods. The spine volume was obtained earlier from a different set of serial sections. Details about how to access the images, data, and programs are available through the OCP ‘DOWNLOAD’ which directs the user to http://w.ocp.me/datum:harris15#download_data.								

**Table 2:** Naming structure and trace type for identification and quantification in Reconstruct and grouped for visualization on OCP visualization

**Objects**	**Distinct Name in Reconstruct**	**Trace Type and OCP characteristics**
***Axons***		Subsequent objects are grouped on the AXONS slider in green color on OCP
stamp	AX##(#)	Stamp identifying an axon as it travels outside the DR volume
excitatory axon	ax##(#)e	Closed trace along membrane of an excitatory axon
inhibitory axon	ax##(#)i	Closed trace along membrane of an inhibitory axon
unidentified axon	ax##(#)u	Closed trace along membrane of an axon not identifiable as excitatory or inhibitory in the volume
axon branch	ax##(#)A,B	Closed trace along membrane of axon branches
myelinated axon	ax##(#)m	Closed trace along membrane of the axon-myelin border
***Dendrites***		Subsequent objects are grouped together on the DENDRITES slider in yellow on OCP
stamp	D##(#)	Stamp used to follow a dendrite outside DR volume
trace	d##(#)	Closed trace along membrane of a spiny dendrite
dendrite branch	d##(#)A, d##(#)B	Closed trace along membrane of a dendrite branch
inerneuron	d##(#)in	Closed trace along membrane of a nonspiny dendritic segment, presumably from interneuron
spinule	d###spinule##	Stamp to identify a spinule
protrusion origins	d##p##	Stamp at base of spines, filopodia, shaft synapses (with 0 protrusion length) on example dendrites
spine fragment	SP##	Stamp for incomplete spines not followed to a dendrite
spine trace	sp##	Closed trace along membrane of a spine fragment
***Synapses***		Subsequent objects are grouped together on the SYNAPSES slider in red on OCP
flat area	d##cfa##Ax##(#)(i,e), sp##cfa##Ax##(#)(i,e); (a,b,c…)	Open and closed traces for PSD area quantification, "Ax##" suffix indicates presynaptic axon. (i=inhibitory, e=excitatory), (a,b,c… unique name for multiple synapses same spine or different heads of a branched spine)
3D visualization	d##c##Ax##(#)(i,e), sp##c##Ax##(#)(i,e); (a,b,c…)	Closed trace encircling a synapse for 3D visualization. Same names as for flat areas.
***Glia***		Subsequent objects are grouped together on the GLIA slider in blue on OCP
astroglia	ag	Closed trace along membrane of astroglia
microglia	mg#	Closed trace along membrane of microglia, # indicates multiple mg
oligodendrocyte	og	Closed trace along membrane of oligodendrocyte
myelin	my##(#)	Closed trace along membrane of myelin sheath, ##—axon number
***Volume and Calibration***		Only in Reconstruct, not included on an OCP slider
cube	cube	Boundary of densely reconstructed volume (DR) of spine and apical dendrite volumes
cylinder	Cyl	Boundary of DR oblique dendrite volume
cylinder line	CylLine	Cross-hairs to center DR on central dendrite
scale	scale	Scale bar for 2D EM, or 3D cubes
mitochondria diameters	mitodia##	Mitochondria diameters for section thickness calculations

**Table 3:** Subcellular components traced in portions of D008 and D000A-B of apical dendrite volume

**Reconstruct Objects**	**Reconstruct Name**	**Trace Type**	**number**
*OCP Slider: Smooth Endoplasmic Reticulum and Spine Apparatus*			
SER-continuous	d###ser	Closed trace along membrane of smooth endoplasmic reticulum	2
spine apparatus	d###sa(n,h)##	Stamp identifying a spine apparatus (n=neck, h=head)	1
SER-like objects	d###ser-like	Stamp identifying SER not obviously distinguishable from an endosome tubule	13
			
*Polyribosomes OCP Slider*			
polyribosome	d###r(s,b,n,h)##	Stamps identifying polyribosomes (s=shaft, b=base, n=neck, h=head)	13
			
*OCP Slider: Mitochondria and microtubules*			
mitochondria	d###mito##	Closed trace along outer membrane of a mitochondria	5
microtubule	d###mt##	Stamp identifying a microtubule in a dendritic shaft	211
			
*OCP Slider: Endosomal Compartments*			
amorphous vesicle	d###av##	Closed trace along membrane of a vesicle of irregular shape	2
autophagosome	d###autophagosome##	Stamp identifying an autophagosome	1
coated pit	d###cp##	Stamp identifying a coated pit	2
coated vesicle	d###cv##	Closed trace along membrane of a coated vesicle	1
larger vesicle	d###ves##	Closed trace along membrane of a larger vesicle	1
lysosome	d###lys##	Closed trace along membrane of a lysosome	1
multivesicular body	d###mvb##	Closed trace along outer membrane of a multivesicular body (mvb)	2
mvb vesicles	d###mvb##v##	Stamp identifying vesicle within multivesicular body	62
small vesicle	d###sv##	Closed trace along membrane of a small round clear vesicle	15
tubule	d###te##	Closed trace along membrane of a tubule	3
vacuole	d###vac##	Closed trace along membrane of a vacuole	1
The OCP Slider combines subsets of these subcellular components as listed.			

**Table 4:** Number of objects in each densely reconstructed volume

**Object Name**	**Number of objects in each densely reconstructed volume.**				**Slider in Open Connectome Project**
	**Names**	**Spine**	**Oblique**	**Apical**	
Excitatory Axons	ax##(#)e	18	97	278	Axons
Inhibitory Axons	ax##(#)i	1	3	8	Axons
Unidentified Axons	ax##(#)u	37	59	115	Axons
Dendrites	d##(#)	8	20	41	Dendrites
Excitatory Synapses	d##(#)c##Ax##(#)e	17	161	488	Synapses
Inhibitory Synapses	d##(#)c##Ax##(#)i	0	4	13	Synapses
Partial Astroglial Processes	ag	many	many	many	Glia
Other Glial Components	og, mg#, my##(#)	1	1	2	Glia
